# Integrative Bioinformatics Analysis Reveals Potential Target Genes and TNFα Signaling Inhibition by Brazilin in Metastatic Breast Cancer Cells

**DOI:** 10.31557/APJCP.2020.21.9.2751

**Published:** 2020-09

**Authors:** Adam Hermawan, Herwandhani Putri

**Affiliations:** 1 *Laboratory of Macromolecular Engineering, Department of Pharmaceutical Chemistry, Faculty of Pharmacy, Universitas Gadjah Mada Sekip Utara II, 55281 Yogyakarta, Indonesia. *; 2 *Cancer Chemoprevention Research Center, Faculty of Pharmacy, Universitas Gadjah Mada Sekip Utara II, 55281 Yogyakarta, Indonesia. *

**Keywords:** Metastasis, breast cancer, bioinformatics, TNFα signalling, Brazilin

## Abstract

**Objective:**

Metastasis is the most significant cause of morbidity and mortality in breast cancer patients. Previously, a combination of brazilin and doxorubicin has been shown to inhibit metastasis in HER2-positive breast cancer cells. This present study used an integrative bioinformatics approach to identify new targets and the molecular mechanism of brazilin in inhibiting metastasis in breast cancer.

**Methods:**

Cytotoxicity and mRNA arrays data were retreived from the DTP website, whereas genes that regulate metastatic breast cancer cells were retreived from PubMed with keywords “breast cancer metastasis”. Gene ontology (GO), Kyoto Encyclopedia of Genes and Genomes (KEGG) pathway enrichment, and Drug association analysis were carried out by using WEB-based GEne SeT AnaLysis Toolkit (WebGestalt). Construction of protein-protein interaction (PPI) network analysis was performed by STRING-DB v11.0 and Cytoscape, respectively. The genetic alterations of the potential therapeutic target genes of brazilin (PB) were analyzed using cBioPortal.

**Results:**

Analysis of cytotoxicity with the public database of COMPARE showed that brazilin exerts almost the same cytotoxicity in the NCI-60 cells panel showing by similar GI50 value, in which the lowest GI50 value was observed in MDA-MB 231, a metastatic breast cancer cells. KEGG enrichment indicated several pathways regulated by brazilin such as TNF signaling pathway, cellular senescence, and pathways in cancer. We found ten drugs that are associated with PB, including protein kinase inhibitors, TNFα inhibitors, enzyme inhibitors, and anti-inflammatory agents.

**Conclusion:**

In conclusion, this study identified eight PB, including *MMP14, PTGS2, ADAM17, PTEN, CCL2, PIK3CB, MAP3K8, *and *CXCL3*. In addition, brazilin possibly inhibits metastatic breast cancer through inhibition of TNFα signaling. The study results study need to be validated with *in vitro* and *in vivo* studies to strengthen scientific evidence of the use of brazilin in breast cancer metastasis inhibition.

## Introduction

Metastasis is the most significant cause of morbidity and mortality in breast cancer patients (Yousefi et al., 2018). The first-line treatment for metastases in breast cancer is chemotherapy (Telli and Carlson, 2009); however, its prolonged use induces side effects, such as cardiotoxicity (Shafei et al., 2017) and suppression of immune system (Larsson et al., 2019). Moreover, the effectivity of chemotherapy in breast cancer treatment decrease due to intrinsic and acquired chemoresistance, which leads to relapse and metastasis (Rivera and Gomez, 2010). Developing a strategy that can overcome the progression and metastasis of breast cancer cells is necessary, including the development of new drugs from natural products.

One compound that has the potential to inhibit metastasis is brazilin, a compound isolated from sappan wood (*Caesalpinia sappan* L.) (Nirmal et al., 2015). Brazilin was shown to induce apoptosis and inhibit cell proliferation in several cancer cells, including MCF-7 breast cancer cells (Naik Bukke et al., 2018), T24 bladder cancer urinary cells (Zhang et al., 2015), U266 multiple myeloma cells (Kim et al., 2012), U87 glioma cells (Lee et al., 2013), Cal27 head squamous cell carcinoma cells and neck cancer cells (Lee et al., 2013), MG-63 osteosarcoma cells (Lee et al., 2018), and Tca8113 tongue cancer cells (Jia et al., 2019). The combination of brazilin and doxorubicin has also been shown to inhibit metastasis in HER2-positive breast cancer through downregulation of MMP9, MMP2, and Rac1 (Jenie et al., 2018). Breast cancer is a complex disease (Fragomeni et al., 2018) and the mechanisms of metastasis involve many key proteins (Welch and Hurst, 2019), and therefore discover new molecular targets and their molecular changes of brazilin in metastatic breast cancer cells needs to be done.

**Figure 1:**
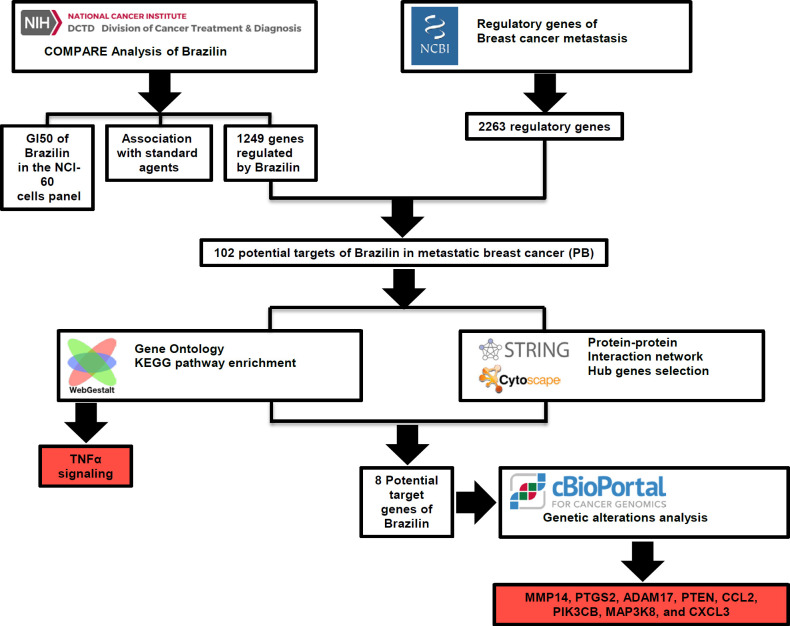
Flowchart of the Study

This present study used an integrative bioinformatics approach to identify new targets and molecular mechanisms of brazilin in inhibiting metastases in breast cancer. The brazilin target was retrieved from the NCI COMPARE, while the metastatic breast cancer regulatory gene was downloaded from PubMed, which from both we made a Venn diagram consisting of the potential therapeutic target of brazilin against metastatic breast cancer cells (Figure 1). Analysis of protein-protein interaction network, gene ontology, enrichment of the KEGG pathway, and genetic alterations, reveal the targets and molecular mechanisms of brazilin in inhibiting metastatic breast cancer. This present study could be the basis for the development of brazilin as an antimetastatic agent in breast cancer.

## Materials and Methods

*Data mining and processing*

 Cytotoxic effect and mRNA expression data were collected from the NCI 60 DTP website (http.dtp.nci.nih.gov) (Monks et al., 1997). COMPARE analysis with the public library generates a list of high similarity drugs with brazilin, and a list of mRNA expressions upon brazilin treatment in the NCI 60 cells collection (Mahmoud et al., 2018). The similarity pattern is presented as the Pearson correlation coefficient, with the cutoff value of <-0.3 and> 0.3. Regulatory genes of metastatic breast cancer were retreived from PubMed database with keywords “breast cancer metastasis“.

*Gene ontology and KEGG pathway enrichment analysis*

Gene ontology (GO) and Kyoto Encyclopedia of Genes and Genomes (KEGG) pathway enrichment analysis were carried out by using Overrepresentation Enrichment Analysis (ORA) from WEB-based GEne SeT AnaLysis Toolkit (WebGestalt), (Wang et al., 2017), with p<0.05 as the cutoff value. 

*Drug association analysis*

Drug association analysis was performed using Overrepresentation Enrichment Analysis (ORA) from WEB-based GEne SeT AnaLysis Toolkit (WebGestalt) with p<0.05 and FDR<0.05 was selected as the cutoff value (Wang et al., 2017). 

*PPI network and hub genes analysis*

PPI network was analyzed with STRING-DB v11.0 (Szklarczyk et al., 2015), with confidence scores >0.7 were considered significant. PPI network further was visualized by Cytoscape. Selection of hub genes based on the highest degree score was conducted by CytoHubba plugin (Chin et al., 2014).

*Analysis of genetic alterations of the PB*

The genetic alterations of the potential therapeutic target genes of brazilin (PB) were analyzed using cBioPortal (Cerami et al., 2012; Gao et al., 2013). The breast cancer study with the highest genetic alterations was chosen for further connectivity analysis with cutoff value of p < 0.05.

## Results

*Data mining and analysis*

**Figure 2:**
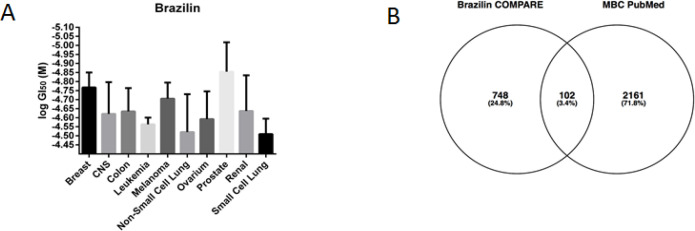
(A). Cytotoxicity (GI50 value) of brazilin in the NCI-60 cells. (B). A Venn diagram between the mRNA array of brazilin (from COMPARE) and genes related to breast cancer metastasis (from PubMed). (C). GO enrichment analysis of Brazilin targets in metastatic breast cancer, analyzed by WebGestalt

This present study aimed to identify the new molecular targets and mechanism of brazilin in inhibition of metastastic breast cancer cells. Analysis of cytotoxicity with the public database of COMPARE showed that brazilin exerts almost the same cytotoxicity in the NCI-60 cells panel showing a similar GI50 value (Figure 2A). The lowest GI50 value was observed in prostate and breast cancer. Moreover, the GI50 value of brazilin in MDA-MB 231, metastatic breast cancer cells, was one of the lowest amongst other cell lines (Supplementary Table 1). 

Analysis with COMPARE identified 13 standard agents with a correlation with brazilin, either direct or inverse (Supplementary Table 2). Pancratiastatin and S-trityl-L-cysteine are standard drugs with the highest score of the Pearson correlation coefficient, whereas AT-125 (acivicin) and mitindomide are standard drugs with the lowest score of Pearson correlation coefficient (Supplementary Table 2). COMPARE analysis revealed 1249 genes regulated by brazilin (Supplementary Table 3), including 587 and 662 genes with the positive and negative Pearson correlation coefficient, respectively. *GLRX3, SMG5, SLC6A4* (with Pearson correlation coefficient of 0.476, 0.459, 0.455, respectively) are genes with direct correlation.* SLC7A11, IL37,* and *PHKB*, with a Pearson correlation coefficient of -0.592, -0.551, and -0.547, respectively, are genes with inverse correlation. Direct correlation shows that the higher mRNA expression, the higher the chemoresistance, while inverse correlation shows that the higher expression of mRNA, the higher the chemosensitivity of the drugs. 

Since brazilin exerted high cytotoxicity towards metastatic breast cancer cells, we retrieved 2,263 regulatory genes of metastasis in breast cancer (Figure 2B) Moreover, a Venn diagram between Brazilin target from COMPARE and regulatory genes of metastatic breast cancer, revealed 102 genes, which were then considered as potential targets of brazilin in metastatic breast cancer (PB).

*GO, KEGG pathway, and drug association analysis of potential targets of brazilin in metastatic breast cancer*

**Figure 3:**
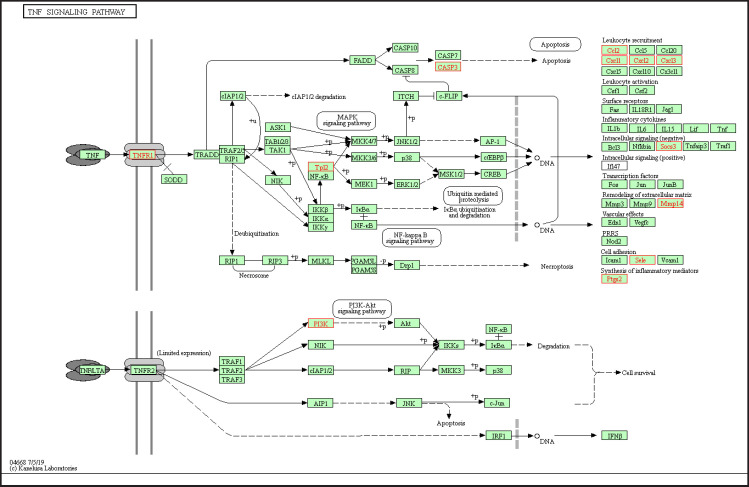
TNF Signaling Pathway (Retrieved from KEGG) as One of the Pathways of Brazilin Targets in Metastatic Breast Cancer

Gene ontology analysis was categorized into biological process, cellular component, and molecular function (Figure 2C). We found that PB was mostly involved in response to stimulus, metabolic process, and cell communication. In addition, the PB was located in the membrane, nucleus, and cytosol, and play a role in the molecular function in protein, ion and nucleic acid binding, as well as enzyme regulator activity. KEGG enrichment indicated several pathways regulated by brazilin (Table 2) such as the TNF signaling pathway, cellular senescence, and pathways in cancer. Several PB was involved in TNFα signaling pathway, including *CASP3, CCL2, CXCL3, MAP3K8, MMP14, PIK3CB, PTGS2,* and *TNFRSF1A* (Supplementary Table 5, Figure 3). From drug association analysis, we found ten drugs that are associated with brazilin, including protein kinase inhibitors, TNFα inhibitors, enzyme inhibitors, and anti-inflammatory agents (Table 2). 

*PPI network and hub genes analysis*

**Figure 4:**
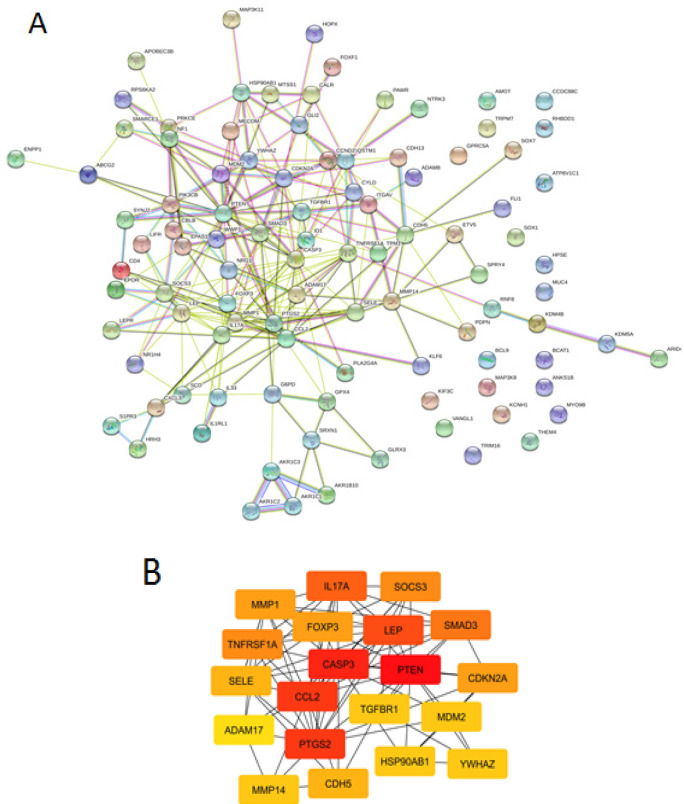
(A). Protein-protein interaction network of Brazilin targets in metastatic breast cancer, analyzed by STRING. (B). Top 20 hub genes based on highest degree score, analyzed by Cytohubba

A PPI network were constructed from 102 proteins (confidence level of 0.4) consists of 99 nodes, 233 edges, PPI enrichment value of <1.10e-16, and average local clustering coefficient of 0.396) (Figure 4A). The top 20 of highest degree score genes were revealed, including, PTEN, CASP3, PTGS2, and ADAM17 (Figure 4B, Table 3).

*Analysis of genetic alterations of the PB*

**Figure 5:**
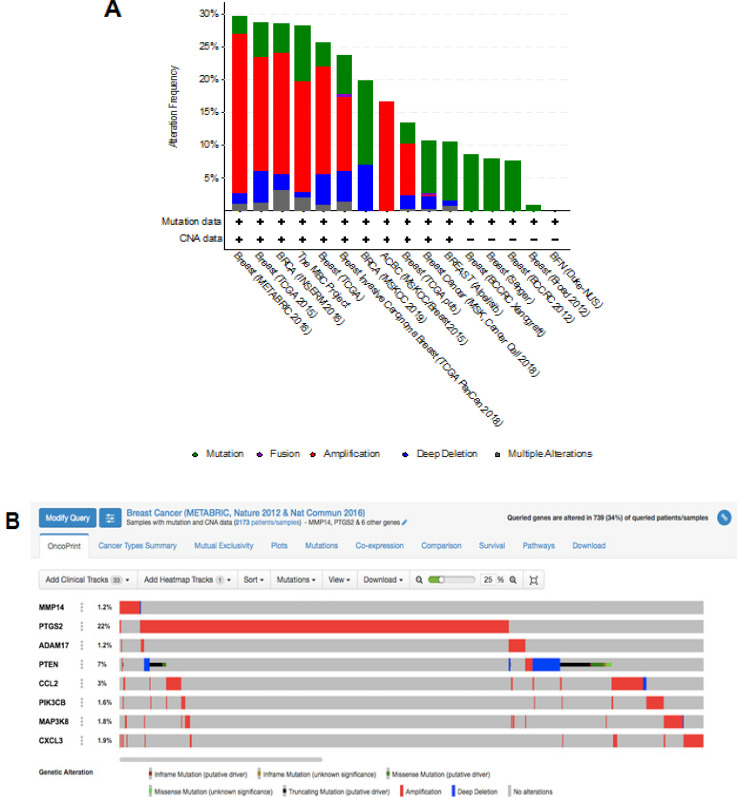
(A). Overview of genetic changes in *MMP14, PTGS2, ADAM17, PTEN, CCL2, PIK3CB, MAP3K8*, and *CXCL3* across 16 breast cancer studies, as analyzed by cBioportal. (B). Summary of alterations in *MMP14, PTGS2, ADAM17, PTEN, CCL2, PIK3CB, MAP3K8,* and* CXCL3* across breast cancer patients using a study from Pereira et al., (2016)

Eight potential target genes of brazilin (PB) were analyzed using cBioportal to explore their genomic alterations across breast cancer studies.* CCL2, MAP3K8, MMP14, PIK3CB*, and *CXCL3* were selected from KEGG pathway enrichment analysis. *PTGS2, MMP14, ADAM17, PTEN, CCL2,* and* CXCL3* were selected based on the highest degree score using CytoHubba. A study, namely the METABRIC (Lefebvre et al., 2016), was selected for further analysis (Figure 5A). Genetic alterations for each target genes were found as *MMP14* (1.2%), *ADAM17* (1.2%),* PIK3CB *(1.6%), *MAP3K8* (1.6%), *CXCL3* (1.9%), *CCL2* (2%), *PTEN* (7%), and *PTGS2* (22%) (Figure 5B). 

**Table 1:** KEGG Pathway Enrichment Analysis of the DEGs

Gene Set	Description	P Value	FDR
hsa04668	TNF signaling pathway	1.02E-07	0.000033174
hsa04218	Cellular senescence	0.000025109	0.0025615
hsa04060	Cytokine-cytokine receptor interaction	0.000026574	0.0025615
hsa04657	IL-17 signaling pathway	0.000033405	0.0025615
hsa05200	Pathways in cancer	0.000039287	0.0025615
hsa04933	AGE-RAGE signaling pathway in diabetic complications	0.000050078	0.0027209
hsa05418	Fluid shear stress and atherosclerosis	0.000059126	0.0027536
hsa05220	Chronic myeloid leukemia	0.000096584	0.003861
hsa05206	MicroRNAs in cancer	0.00010659	0.003861
hsa05163	Human cytomegalovirus infection	0.00034585	0.011275
hsa05142	Chagas disease (American trypanosomiasis)	0.00048594	0.014402
hsa04659	Th17 cell differentiation	0.00062734	0.014837
hsa04931	Insulin resistance	0.00062734	0.014837
hsa04932	Non-alcoholic fatty liver disease (NAFLD)	0.00063716	0.014837
hsa04115	p53 signaling pathway	0.00069172	0.015033
hsa04390	Hippo signaling pathway	0.00077556	0.015802
hsa04217	Necroptosis	0.0010456	0.018937
hsa04630	JAK-STAT signaling pathway	0.0010456	0.018937
hsa04380	Osteoclast differentiation	0.0015995	0.027443
hsa04068	FoxO signaling pathway	0.0018718	0.030511
hsa05222	Small cell lung cancer	0.0020866	0.032393
hsa04010	MAPK signaling pathway	0.0023689	0.035103
hsa05215	Prostate cancer	0.0026322	0.037308
hsa05161	Hepatitis B	0.002903	0.039432
hsa04625	C-type lectin receptor signaling pathway	0.0035609	0.046434
hsa05230	Central carbon metabolism in cancer	0.0037962	0.047599

**Table 2:** Top 10 Drug Association Analysis

Gene Set	Description	P value	FDR
PA164712838	Interleukin inhibitors	6.63E-11	1.21E-07
PA164713204	Protein kinase inhibitors	3.57E-09	0.000003264
PA164713366	Tumor necrosis factor alpha (TNF-alpha) inhibitors	7.16E-08	0.000037631
PA166049190	flufenamic acid	8.24E-08	0.000037631
PA164712732	Enzyme inhibitors	1.08E-07	0.000039341
PA164712734	Enzymes	1.71E-07	0.000051958
PA164712839	Interleukins	1.76E-06	0.00045974
PA164774763	latanoprost	0.000016719	0.0038181
PA164712458	Antiinflammatory Agents	0.000021643	0.0043934
PA450744	Oxygen	0.000027381	0.0044111

**Table 3:** Top 20 Hub Genes Based on Highest Score Degree, Analyzed by CytoHubba

Rank	Gene Symbol	Gene Name	Score
1	PTEN	Phosphatase and tensin homolog	26
2	CASP3	Caspase-3	25
3	PTGS2	Prostaglandin G/H synthase 2	19
3	CCL2	C-C motif chemokine 2	19
5	LEP	Leptin	16
6	IL17A	Interleukin-17A	15
7	SMAD3	Mothers against decapentaplegic homolog 3	14
8	TNFRSF1A	Tumor necrosis factor receptor superfamily member 1A	13
8	SOCS3	Suppressor of cytokine signaling 3	13
10	FOXP3	Forkhead box protein P3	12
10	MMP1	Interstitial collagenase	12
10	CDKN2A	Cyclin-dependent kinase inhibitor 2A	12
13	CDH5	Cadherin-5	10
13	SELE	E-selectin	10
15	HSP90AB1	Heat shock protein HSP 90-beta	9
15	TGFBR1	TGF-beta receptor type-1	9
15	MDM2	E3 ubiquitin-protein ligase Mdm2	9
15	MMP14	Matrix metalloproteinase-14	9
15	YWHAZ	14-3-3 protein zeta/delta	9
20	ADAM17	Disintegrin and metalloproteinase domain-containing protein 17	8

## Discussion

This study was aimed to identify the new targets and molecular mechanisms of brazilin in inhibition of metastases in breast cancer using an integrative bioinformatics approach. Analysis using COMPARE showed that brazilin had the lowest GI50 value amongst other cells in MDA-MB 231 cells, highly metastatic breast cancer cells (Liu et al., 2019). This phenomenon revealed the high potency of brazilin as an anticancer agent against metastatic breast cancer.

Analysis with COMPARE identified 13 standard agents that have a correlation with brazilin, in which pancratiastatin and S-trityl-L-cysteine are standard drugs with the highest score of Pearson correlation coefficient, whereas AT-125 (acivicin) and mitindomide are standard drugs with the lowest score of Pearson correlation coefficient. The high value of the Pearson correlation coefficient means, the higher the cytotoxicity of a standard compound, the higher the cytotoxicity of brazilin. Vice versa, the low value of the Pearson correlation coefficient means the higher the cytotoxicity of a standard compound, the lower the cytotoxicity of brazilin. A previous study showed that pancratistatin, alkaloid compound isolated from Amaryllidaceae plant, promotes apoptosis and autophagy in DU145 and LNCaP metastatic prostate cancer cells (Griffin et al., 2011). Another study demonstrated that S-trityl-L-cysteine isolated from garlic is a novel Eg5 inhibitor for chemotherapy against neuroblastoma cells (Wu et al., 2018). Acivicin, an antibiotic, and chemotherapy isolated from Streptomyces sviceus (Poster et al., 1981). A phase II clinical trial of acivicin has been performed against advanced metastatic breast cancer patients (Fleishman et al., 1983). Moreover, a combination of acivicin and cisplatin inhibits metastasis in B16F10 melanoma cells (Roy and Maity, 2007). Another study showed that mitindomide free and liposomal drugs showed cytotoxicity against L1210 leukemic cells (Sampedro et al., 1994). Moreover, mitindomide exerts cytotoxicity through inhibition of topoisomerase II (Hasinoff et al., 1997). Accordingly, brazilin probably acts as pancriastatin in inhibiting metastatic breast cancer cells.

KEGG pathway enrichment analysis revealed that brazilin targets the TNF signaling pathway in inhibition of metastatic breast cancer cells. Tumor necrosis factor-alpha (TNFα) is a cytokine that regulates various biological processes of cancer, including inflammation (Scheff et al., 2017), cell proliferation and apoptosis (Lyu et al., 2017), progression and metastasis (Ham et al., 2016), and chemoresistance (Zhang et al., 2018). The signaling starts when TNFα binds into its receptor, TNFαRI (a member of TNFαR family together with TNFαRII), and leads to receptor trimerization and the recruitment of adaptor protein and TNFαR associated factor, followed by activation of IKK and transactivation of NFkB (Wu and Zhou, 2010). 

TNFα signaling pathway regulates metastasis in breast cancer through several mechanisms. Activation of NFkB by TNFα promotes breast cancer by promoting cell proliferation and migration in breast cancer cells (Kawabata et al., 2017). TNFα signaling also activates the ERK/JNK/p38 pathway leading to migration and invasion in colon cancer cells (Zhao and Zhang, 2018), and breast cancer cells (Qiu et al., 2019). Another study demonstrated that TNFα activates mesenchymal stromal cells that leads to metastasis in breast cancer through the recruitment of CXCR2+ neutrophils (Yu et al., 2017). In addition, TNFα contributes to the aggressive properties of triple-negative breast cancer cell lines via the upregulation of TNFAIP3(A20) (Lee et al., 2019). Recently, TNFα involves in the invasion and metastasis of breast cancer cells by promoting crosstalk between mitochondria and lysosomal function (Singh et al., 2019). The results of KEGG pathway enrichment analysis is supported by the results of drug association analysis, which showed that potential therapeutic target of brazilin against metastatic breast cancer cells (PB) are associated with tyrosine kinase inhibitors and TNFα inhibitors (Supplementary Table 6). Nevertheless, these findings need to be validated further with in vitro and in vivo studies. 

In the following section, we will discuss each gene target, its potential as a target of brazilin, and its relationship to TNFα signaling. MMP14 encodes matrix metalloproteinase 14 (Mmp14) or also known as membrane type-1 matrix metalloproteinase (MT1-MMP), an enzyme that plays a role in the breakdown of extracellular matrix in invasion, metastasis, and angiogenesis (Shuman Moss et al., 2012). Overexpression of* MMP14* in serum was found in patients with invasive gastric cancer (Kasurinen et al., 2018). A recent study revealed that MMP14 is a biomarker of poor prognosis in patients with colorectal cancer (Cui et al., 2019). MMP19 plays a role in the TNFα signaling pathway. TNFα increased the expression of MMP14 during fracture healing (Lehmann et al., 2005). Moreover, TNFα activates the MAPK/ERK signaling pathway and subsequently promotes the migration of breast cancer cells through the upregulation of MMP9, MMP14, and MMP2 in the lipid rafts (Wolczyk et al., 2016). Nevertheless, the role of MMP14 and TNFα signaling upon brazilin treatment needs to be explored further.

*PTGS2* encodes prostaglandin-endoperoxide synthase 2 or known as cyclooxygenase 2 (COX2), a key regulatory enzyme in the biosynthesis of prostaglandin E2 (Kozak et al., 2000). Overexpression of COX2 was found in about 40% of patients with invasive breast cancers (Singh et al., 2006). COX2 promotes bone metastasis of breast cancer cells through the upregulation of Interleukin-11 (IL-11) (Singh et al., 2006). Previous studies showed that COX2 promotes metastasis in breast cancer cells through several mechanisms, including infiltration by regulatory T cells (Tregs) (Karavitis and Zhang, 2013) and activation of the VEGF signaling pathway to trigger angiogenesis (Xu et al., 2014). 

The crosstalk between COX2 and TNFα was discussed in several previous studies. A previous study showed that TNFα induces the COX2 expression via activation of the NFkB signaling pathway in human colon cancer cells (Plummer et al., 1999), and activation of P38/MAPK pathway in colonic myofibroblast (Saini et al., 2016). TNFα also promotes migration in colonic myofibroblast via activation of COX2 that is mediated by the P38/MAPK pathway (Saini et al., 2016). Moreover, TNFα also induces COX2 expression and subsequently increased PGE2 production and MMP9 expression in human fibroblast-like synovial cells (Alsousi et al., 2017).

*ADAM17* encodes ADAM metallopeptidase domain 17, a member of a disintegrin and metalloprotease domain family, and is also known as tumor necrosis factor (TNF)-converting enzyme (Li et al., 2019c). A previous study demonstrated that ADAM17 plays a role in the shedding of TNFα and membrane receptors protein, which involved in the biological process of proliferation, migration, and differentiation (Gutiérrez-López et al., 2011). On the other hand, ADAM17 is a pivotal enzyme for the generation of TNF-α in oral keratinocytes (Hirayama et al., 2017), and hemophilic arthropathy (Haxaire et al., 2018). Overexpression of ADAM17 promotes 5-fluorouracil resistance and metastasis in colorectal cancer cells via activation of the Notch signaling pathway (Li et al., 2018), and induces metastasis in gastric cancer through activation of Notch and Wnt signaling pathways (Li et al., 2019c). A review article discussed ADAM17 inhibitors for inflammation and cancer therapy (Kanda et al., 2017). However, the role of brazilin in ADAM17 activity and TNFα in promoting metastasis need to be clarified further. 

*PTEN* encodes a phosphatidylinositol-3,4,5-trisphosphate 3-phosphatase, a negative regulator of PI3K/Akt signaling pathway (Maehama et al., 2001). A previous study revealed the association between the loss of PTEN and metastasis in patients with endometrial cancer (Salvesen et al., 2002), prostate and breast cancer cells (Bandyopadhyay et al., 2004). Previous studies revealed the role and mechanisms of PTEN in breast cancer metastasis. PTEN inhibits proliferation, migration, and invasion in osteosarcoma cells by downregulating MMP9 and focal adhesion kinase (FAK) (Hu et al., 2014). Another study demonstrated that PTEN hampers invasion and metastasis of gastric cancer cells through the inhibition of the PI3K/NFkB pathway and preventing the DNA binding of NFkB on the FAK promoter (Zhang et al., 2014). Moreover, PTEN inhibits metastasis in breast cancer cells through the downregulation of WNT1 inducible signaling pathway protein 1 (WISP1) and lipocalin-2 (LCN2) (Chiang et al., 2016), and inactivation of NFkB signaling in non-small cell lung cancer cells (Akgun et al., 2019). A previous study showed that TNFα increased the expression of PTEN through activation of NFkB signaling in HL60 human leukemic cells (Lee et al., 2007). However, other studies demonstrated that PTEN inhibited the PI3K/AKT/NFκB signaling pathway that is induced by TNFα in human glioma cells (Koul et al., 2006), and prostate cancer cells (Lee et al., 2007). Further study of brazilin on the axis of PTEN-NFkB-TNFα in metastatic breast cancer cells is required.

The results of this present study showed genetic alterations in 7% of patients with metastatic breast cancer in the METABRIC study (Figure 4B). This result is supported by a recent study which demonstrated that mutation in *PTEN* leads to inactivation of its function as tumor suppressor genes in cancer (Luongo et al., 2019). A recent review article discussed the possibility of PTEN as a potential biomarker of lymph node metastasis of esophageal squamous cell carcinoma (Li et al., 2019b). Taken together, PTEN plays a pivotal role in breast cancer metastasis. 

*CCL2* encodes C-C motif chemokine ligand 2, also known as monocyte chemoattractant protein 1 (MCP1), a chemokine that regulates inflammatory processes (Daly and Rollins, 2003). Recent studies showed that CCL2 also regulates cancer metastasis. A previous study showed that CCL2 induces chemokine cascade signaling and subsequently promotes breast cancer metastasis by elevating retention of metastasis-associated macrophages (Kitamura et al., 2015). Another study demonstrated that CCL2 plays a role in macrophage recruitment that regulates lymphatic metastasis of bladder cancer (Chen et al., 2018). Increased CCL2 secretion was also found in the protumoral microenvironment induced by retinoblastoma inactivation (Li et al., 2019a). 

The axis between TNFα signaling and CCL2 has also been studied, in which TNFα increases the expression of CCL2 in human proximal tubular epithelial cells via activation of MAPK signaling (Ho et al., 2008). A recent clinical trial of an inhibitor of CCL2, namely propagermanium, showed proper safety and efficacy in patients with metastatic breast cancer (Masuda et al., 2020). Taken together, CCL2 is a promising target for inhibiting breast cancer metastasis; however, the role of brazilin in metastasis-related to CCL2 and TNFα signaling needs to be explored further. 

PIK3CB encodes phosphatidylinositol-4,5-bisphosphate 3-kinase catalytic subunit beta (PI3Kβ), also known as p110β, a protein involved in the PI3K/Akt signaling pathway (Pridham et al., 2018). Overexpression of PIK3CB was found in patients with colorectal carcinoma (Wen et al., 2014), non-small cell lung cancer (Xiong et al., 2017). Inhibition of PIK3CB with a specific inhibitor, reduced cell viability, and proliferation in glioblastoma (Pridham et al., 2018). In addition, PIK3CB, a significant regulator of the PI3K/Akt pathway, regulates metastasis in breast cancer cells (Hong et al., 2019). The PI3K/Akt signaling is involved in the TNFα pathway in airway remodeling (Jude et al., 2012). Activation of PI3K/Akt signaling increased the secretion of TNFα in activating macrophages (Huang et al., 2013). Nevertheless, the effects of brazilin on PIK3CB in metastatic breast cancer cells remain unclear.

*MAP3K8* encodes mitogen-activated protein kinase kinase kinase 8, a member of serine/threonine kinase family (Paardekooper et al., 2018), which is involved in the MAPK/JNK/p38 and NFkB signaling pathway (Chorzalska et al., 2018). Overexpression of MAP3K8, also known as tumor progression locus 2 (TPL2), is correlated with poor prognosis and metastasis in patients with colorectal cancer (Pyo et al., 2018). Another study demonstrated that MAP3K8 induces invasion and metastasis in renal cancer cells (Liu et al., 2016). MAP3K8 was also found to involve in the maintenance of thyroid cancer stem cells and thyroid cancer resistance to vemurafenib (Gianì et al., 2019). Genetic alterations in MAP3K8, including fusion and truncation, were found in 33% of patients with Spitz melanoma (Newman et al., 2019). Regarding the crosstalk with TNFα signaling, MAP3K8 was found to increase the expression of TNFα in monocyte-derived dendritic cells (Paardekooper et al., 2018). Accordingly, the role of MAP3K8 in metastatic breast cancer and the effect of brazilin on MAP3K8 need to be explored in the future study.

*CXCL3* encodes C-X-C motif chemokine ligand 3, a member of the CXC subfamily of chemokines, also known as a growth-related oncogene, is a potent neutrophil chemoattractant that regulates inflammation (Smith et al., 2005). The upregulation of CXCL3 was found in metastatic breast cancer and posses a potential therapeutic target (See et al., 2014). Other studies showed that CXCL3 is involved in migration and invasion of trophoblasts in the pathogenesis of preeclampsia (Wang et al., 2018) and is involved in the proliferation and migration of prostate cancer cells (Xin et al., 2018). A previous study demonstrated that CXCL3 is upregulated upon treatment of TNFα in adipocytes (Kusuyama et al., 2016). In addition, TNFα induced the upregulation of CXCL3 and its receptor in A498 renal cancer cells (Sun et al., 2016). Taken together, CXCL3 and its signaling is a potential target for preventing metastatic breast cancer. However, the effect of brazilin on CXCL3 signaling related to TNFα in metastatic breast cancer remains elusive. 

Previous studies have demonstrated the effect of brazilin on TNFα signaling and PB. Brazilin suppressed the production of TNFα in lipopolysaccharide-induced RAW264.7 macrophages cells (Hu et al., 2009). Brazilin inhibited TNFα-induced NFkB signaling by targeting IKK in 293/IL-1R/TLR4 cells (Jeon et al., 2014). Brazilin was shown to decrease high glucose-induced vascular inflammatory through the inhibition of NFκB activation in HUVEC cells (Jayakumar et al., 2014). Brazilin was also found to decrease the expression of TNFα in mice with type-II collagen-induced arthritis (Jung et al., 2015) and to decrease the expression of COX2 and TNFα, and inhibit ERK/NFkB signaling in RANKL-stimulated RAW264.7 cells (Kim et al., 2015). Recently, a study showed that brazilin possesses anti-inflammatory activity in TNFα-induced psoriasis dermatitis by downregulating ERK/JNK/p38 and NFkB signaling (Choi and Hwang, 2019). Further study of the effects brazilin on TNFα signaling and PB in metastatic breast cancer cells is required. 

This present study revealed the new target genes and the mechanism of brazilin in inhibiting metastasis in breast cancer using a bioinformatics approach. This study identified eight new potential targets of brazilin for inhibiting metastatic breast cancer, including *MMP14, PTGS2, ADAM17, PTEN, CCL2, PIK3CB, MAP3K8*, and *CXCL3*. In addition, brazilin possibly inhibits metastatic breast cancer through inhibition of the TNFα signaling. The study results need to be validated with *in vitro* and *in vivo *studies to strengthen scientific evidence of the use of brazilin in inhibition of breast cancer metastasis.

## Availability of material

The datasets analysed during the present study are online available in the public database.
